# Estimating Real-Time qPCR Amplification Efficiency from Single-Reaction Data

**DOI:** 10.3390/life11070693

**Published:** 2021-07-14

**Authors:** Joel Tellinghuisen

**Affiliations:** Department of Chemistry, Vanderbilt University, Nashville, TN 37235, USA; joel.tellinghuisen@vanderbilt.edu; Tel.: +1-615-322-4873; Fax: +1-615-343-1234

**Keywords:** qPCR, data analysis, nonlinear least squares, statistical errors, calibration

## Abstract

Methods for estimating the qPCR amplification efficiency *E* from data for single reactions are tested on six multireplicate datasets, with emphasis on their performance as a function of the range of cycles *n*_1_–*n*_2_ included in the analysis. The two-parameter exponential growth (EG) model that has been relied upon almost exclusively does not allow for the decline of *E*(*n*) with increasing cycle number *n* through the growth region and accordingly gives low-biased estimates. Further, the standard procedure of “baselining”—separately estimating and subtracting a baseline before analysis—leads to reduced precision. The three-parameter logistic model (LRE) does allow for such decline and includes a parameter *E*_0_ that represents *E* through the baseline region. Several four-parameter extensions of this model that accommodate some asymmetry in the growth profiles but still retain the significance of *E*_0_ are tested against the LRE and EG models. The recursion method of Carr and Moore also describes a declining *E*(*n*) but tacitly assumes *E*_0_ = 2 in the baseline region. Two modifications that permit varying *E*_0_ are tested, as well as a recursion method that directly fits *E*(*n*) to a sigmoidal function. All but the last of these can give *E*_0_ estimates that agree fairly well with calibration-based estimates but perform best when the calculations are extended to only about one cycle below the first-derivative maximum (FDM). The LRE model performs as well as any of the four-parameter forms and is easier to use. Its proper implementation requires fitting to it plus a suitable baseline function, which typically requires four–six adjustable parameters in a nonlinear least-squares fit.

## 1. Introduction

Quantitative polymerase chain reaction (qPCR) is an analytical method for estimating numbers of molecules of specific genetic substances through amplification to easily detected quantities [1]. The standard approach for “absolute” quantification is calibration procedures that compare the unknown with results for the same substance measured at a range of known concentrations chosen to encompass the unknown [2,3]. The standard curve plots of quantification cycle *C_q_* vs. the logarithm of the template number (*N*_0_) are ideally linear, with slope −1/log(*E*), where *E* is the amplification efficiency (AE). Recommendations for producing such curves involve three or more replicates at each of the five–seven concentrations for unknown estimation, or even more concentrations if the AE is to be estimated [4,5,6]. This procedure could then entail at least 15 and as many as ~30 individual PCR reactions, with an estimation of *C_q_* for each.

It has long been a dream to reduce this time and materials demand by estimating the AE directly from the growth profile for a single reaction (SR) [7,8,9]. This would then permit quantitative estimation of the unknown with a single calibration reference, or even from the SR itself, if optical calibration could be trusted [10]. A number of such methods have been discussed in detail and their performance compared on several multireplicate datasets by Ruijter, et al. [11]. Clearly, the value of these methods depends upon their accuracy and precision.

**Figure 1:**
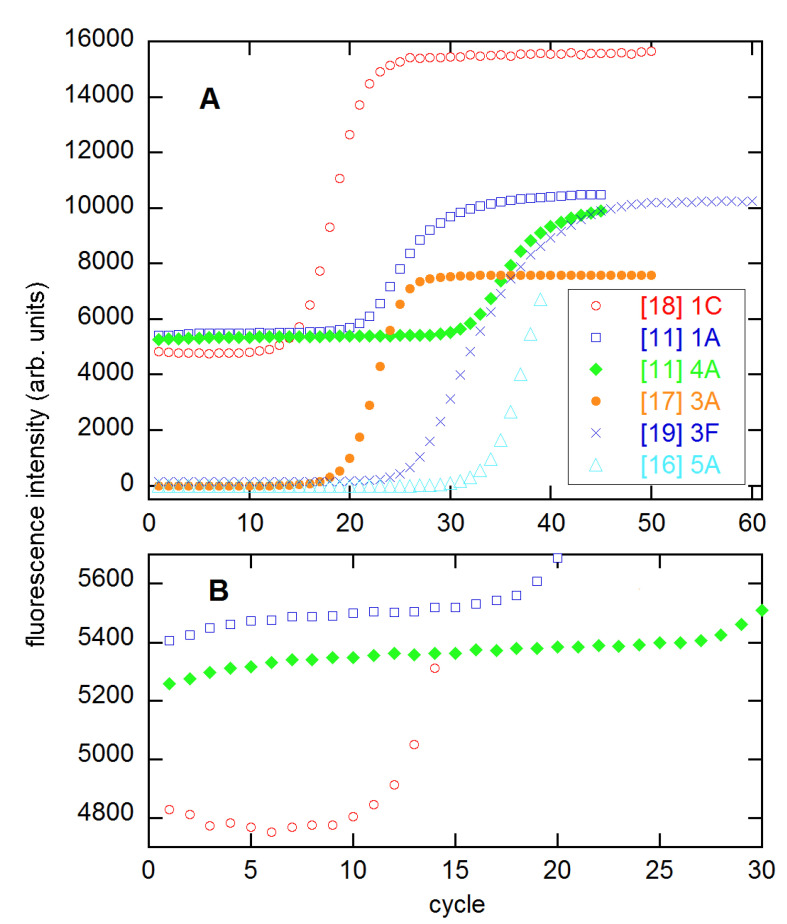
Representative growth profiles (**A**) and expanded baselines (**B**) from the indicated references. Numbers give the concentration, from highest to lowest, and letters represent the replicate of that concentration. The two profiles from [11] are from the 94 × 4 technical replicates set. The profiles from [16,17] have been scaled up by factors of 2000 and 200, respectively, for presentation. These data and those from [3] (not shown) appear to have had baselines subtracted.

In [11], Ruijter and coauthors included much information about accuracy and precision in their comparisons. However, Spiess and I have pointed out statistical weaknesses in the implementations of many of these methods [12,13,14,15]. Moreover, there are other SR methods that were not considered in [11] that might perform better. The topic of the present work is a more detailed examination of such methods, especially including the dependence of the results on the range of cycles *n*_1_–*n*_2_ included in the analysis. To this end, I employ six multireplicate datasets, the same number included in the comparisons of [15], which focused on the estimation of *C_q_* and its use in calibration. These datasets are from [3,11,16,17,18,19], representative profiles from which are shown in Figure 1.

Before getting to the details of the present calculations, it is useful to note the aforementioned statistical weaknesses in many methods. In almost all of those compared in [11], the baseline is estimated and then subtracted before the fluorescence data are analyzed. In fact, data are often *presented* after baselining, as is the case for at least three of the six datasets used here and shown in Figure 1. However if the model for the data is baseline + signal, then the proper statistical analysis should be a direct fit to this model, since the baseline is presumed to contribute to the total signal at all cycles. This requires nonlinear least squares (NLS), but user-friendly programs for NLS have been available for decades. Through Monte Carlo simulations, Spiess and I showed in the Supporting Information to [14] (Figures S1–S5) that this two-step procedure leads to an increase of 75% in the dispersion of AE estimates, which translates into an efficiency loss of 1.75^2^ ≈ 3. This means that one reaction analyzed properly is the statistical equivalent of three analyzed in the two-step procedure. One method covered in [11] that did include a baseline in the fitting was PCR-Miner [20]; in fact, this method won out in the *C_q_* precision comparisons in [12].

A second problem with most SR methods is reliance on the exponential growth model,

*y* = *y*_0_ *E^n^*, (1)

where the AE ranges from *E* = 1 (no amplification) to *E* = 2 (perfect doubling), *n* is the cycle number, and *y* represents the fluorescence signal, with *y*_0_ being that in Cycle 0. However, *E* must decline from ~2 in the baseline region to 1 in the plateau region at large *n*, so the use of Equation (1) for early growth cycles is questionable. In fact, using the LRE model [21] to generate data resembling typical growth profiles, Spiess and I showed that analysis with Equation (1) yields low-biased estimates of *E* when enough cycles are included to give good precision [14]. The extent of that problem for actual data is a major concern of the present work. 

**Figure 2:**
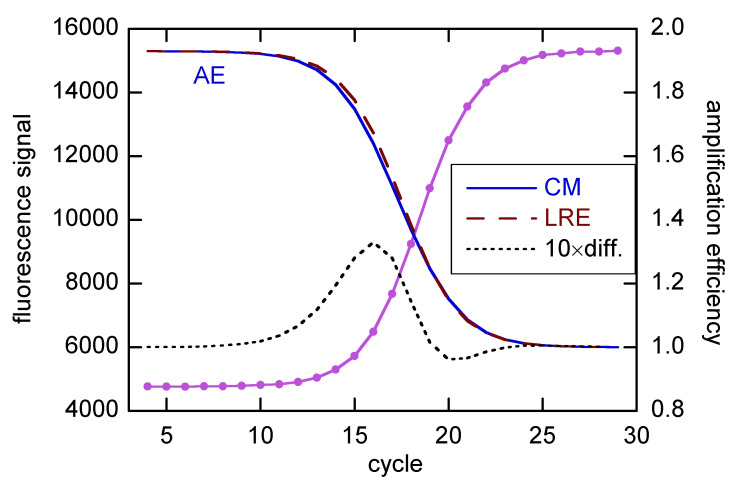
Comparison of CMa and LRE estimates of amplification efficiency (scale to right) for rx std1 from [18], which is an average of rxs 1A-1C. For the purpose of this figure, *E*_0_ for both fits was fixed at 1.93, as obtained for the LRE model (see below). The AE difference (LRE-CM) has been scaled by a factor of 10 and incremented by 1, for display on the same AE grid. The baseline was taken as linear, and the fits were done for Cycles 4–29.

The LRE model contains a parameter (*E*_0_) for the AE in the baseline region, with *E* decreasing properly to 1 in the plateau region. The decline of *E* through the growth region is illustrated in Figure 2, with a comparison with one additional method, from Carr and Moore [22]. The CM method is an extension of the MAK2 method [23] (covered in [11]), but with one additional parameter that allows for the decline of *E*, leading to realistic sigmoidal profiles. Both methods tacitly assume *E* = 2 in the baseline region. However, with a minor modification, *E* can be made an adjustable parameter in the CM method.

The AE from the LRE fit in Figure 2 agrees closely (~0.02) with the calibration estimate [15]. I will use such agreement as the quality marker in the present study, as there seems no better way to judge the SR estimates for quantifying *N*_0_ in unknowns. However, there are reasons the two estimates may not agree. Most importantly, the SR estimates pertain to the early growth region, while the calibration estimates actually cover the early cycles in the baseline region up to the first cycle for the most concentrated calibrant [12]. This follows from the assumption that a dilute reaction amplifies the same as a more concentrated one after *N* for the dilute reaction matches *N*_0_ for the concentrated one. Thus, all of the difference comes from the first Δ*C_q_* cycles of the dilute sample, and that Δ*C_q_* determines *E* for the pair.

In the present work, I emphasize the performance of the simple growth model of Equation (1) and the essentially logistic LRE model. I also consider variations of the latter with an additional parameter to handle asymmetry in the amplification profiles, as well as the CM model and a direct-fitting approach that assumes a logistic *E*(*n*). In general, for the present tests, these methods do not perform as well as just fitting to the LRE model over a limited cycle range, namely, to about the half-intensity point or the first-derivative maximum (FDM). 

## 2. Mathematical Background

The simplest way to estimate the AE from SR data is by fitting to Equation (1), which contains just two parameters apart from those needed to represent the baseline. As already noted, most of the methods reviewed in [11] are variations on this theme, with several ways of treating the baseline, which is estimated separately and subtracted. Some methods select a range of cycles where the logarithmic transformation of the “baseline-corrected” data appears to be linear and then estimate *E* from a linear fit of these transformed data. In testing this model here, which I will call the EG (exponential growth) model, I will employ the statistically best approach: fit the data to Equation (1) plus a baseline function. The issue is then how far into the growth phase to extend this fit, and the usual answer is to about the second derivative maximum (SDM) cycle. I will examine results for fitting to cycle *n*_2_ spanning a range including the SDM.

The LRE *method* devised by Rutledge and Stewart [21] involves several steps. However, the results are equivalent to just fitting to the three-parameter model [24],

(2) $$y(n)=\frac{y_{0}y_{\max}E_{0}^{n}}{y_{0}E_{0}^{n}+y_{\max}-y_{0}}$$

(3) $$\approx \frac{y_{\max}}{1+\exp(b(n_{1/2}-n))}$$

for which I will retain the LRE label. Here, *y*_max_ is the limiting growth and *E*_0_ the initial AE. The second expression is the logistic model and is obtained by neglecting *y*_0_ in the denominator of the first; *n*_1/2_ is the half-intensity point and the FDM, with *b* = ln(*E*_0_) and *y*_max_/*y*_0_ = *E*_0_*n*1/2. Because normally *y*_0_ << *y*_max_, there is insignificant difference in results obtained with Equations (2) and (3). Figure 2 shows that this model can give very good results for *E*(*n*), which is typically found to have declined by about 0.2 at the SDM [14]. However, it is inherently symmetrical about the half-intensity point (also the FDM), which most qPCR curves are not. Accordingly, it does not perform well in whole-curve fitting of asymmetrical growth profiles. Below, I describe how to include an asymmetry parameter to improve the fit quality.

The log-logistic model is a sigmoidal alternative to Equation (3), and in the four-parameter form [25],

LL4(*n*) = *y*_max_ [1 + (*g*/*n*)^*h*^]^−*p*^ (4)

it can accommodate some asymmetry. With *p* = 1, it is nearly symmetrical, and *g* ≈ *n*_1/2_ (≈*n*_FDM_). This model was used in [15] to obtain the most precise *C_q_* estimates, by fitting to typically 22–26 cycles centered on the FDM. It cannot predict *E*_0_ in the baseline region, because there are no parameters for this. However, it is useful for estimating *y*_max_ in calculations where this parameter is held fixed in the LRE models.

### 2.1. The Logistic Model with Asymmetry

One can incorporate asymmetry in the model of Equation (2) by raising the denominator to the power *p*, as in Equation (4). In both cases there are two modes of convergence that are statistically inequivalent. An alternative is to raise *y*_max_ in the denominator of Equation (2) to the power *p* and then raise the entire denominator to the power *p*^−1^. This approach ensures that at small *n*, where *y*_max_ dominates the denominator, *E*_0_ retains its significance as the initial AE, with physically reasonable prediction of *E*(*n*) into the growth region. Similarly, the addition of the asymmetry parameter to Equation (3) preserves its ability to predict physically reasonable *E*(*n*). The issue again is how well do the results agree with calibration-based *E* for asymmetrical profiles?

We want to express the modified versions of Equation (3) in ways that contain *E*_0_ and the FDM as adjustable parameters, so that the LS fits return these and their standard errors (SE) directly. We first note that *n*_1/2_ in Equation (3) *is* the FDM, and by replacing *b* by ln(*E*_0_), we obtain *E*_0_ directly. Taking Mode A as the four-parameter version of Equation (3), we have

(5) $$yA\;(\equiv \mathrm{LREA})=\mathrm{base}(n)+\frac{y_{\max}}{{\left(1+\frac{1}{p}\exp\left[\frac{\ln(E_{0})}{p}(n_{\mathrm{FDM}}-n)\right]\right)}^{p}}$$

In Mode B the sigmoidal function can be expressed as a subtraction from the plateau level [15], but it can also be written as

(6) $$\mathrm{LREB}=\mathrm{base}(n)+y_{\max}\left[1-{\left(1+\frac{1}{p}\exp\left[\ln(E_{0})(n-n_{\mathrm{FDM}})\right]\right)}^{-p}\right]$$

in which form it is added to base(*n*). Mode C is obtained from Equation (2) as already described, by raising *y*_max_ in the denominator to power *p* and the entire denominator to power *p*^−1^:

(7) $$\mathrm{LREC}=\mathrm{base}(n)+\frac{y_{0}y_{\max}E_{0}^{n}}{{\left(y_{0}E_{0}^{n}+y_{\max}{}^{p}-y_{0}\right)}^{1/p}}$$

We obtain a nearly equivalent expression by replacing *y*_0_ as a parameter with the FDM,

(8) $$\mathrm{LRED}=\mathrm{base}(n)+\frac{y_{\max}E_{0}^{n}}{{\left(E_{0}^{n}+{\left(E_{0}^{n_{\mathrm{FDM}}}\right)}^{p}-1\right)}^{1/p}}$$

in the same way that we went from Equation (2) to Equation (3). All of these expressions revert to LRE when *p* = 1. 

### 2.2. Recursion Models

The “mechanistic” models of [22,23] employ recursion relations to generate *y*(*n*) from *y*(*n* − 1), based on elements of the reaction chemistry thought to hold in the PCR process. As already shown in Figure 2, the Carr–Moore (CM) model, with three adjustable parameters, can predict realistic behavior for both the profiles and *E*(*n*) [22]. The MAK2 model of [23], like the EG model of Equation (1), grows without limit, so must be restricted to the early growth region in analysis. Because of these limitations and because the CM model is an extension of it, I do not consider MAK2 any further here.

An alternative recursion approach defines *E*(*n*) as a sigmoidal function, with data fitted to

*y*(*n*) = *y*(*n*−1) *E*(*n*−1) (9)

For the datasets I have analyzed with this method, its performance has been poorer than that of the other approaches, so I provide only limited results for it below.

The recursion relation of the CM model is

(10) $$y_{i}=y_{i-1}\left(1+\frac{A-y_{i-1}}{A}-\frac{y_{i-1}}{K_{d}+y_{i-1}}\right)=y_{i-1}\left[2-y_{i-1}\left(\frac{1}{A}+\frac{1}{K_{d}+y_{i-1}}\right)\right]$$

where I have retained their notation (*i* = *n*) of [22] and have used *A* in place of *y*_max_, because I have found that *A* does not have the physical significance of *y*_max_, typically being a factor of 3 larger than the plateau. The final version of the relation makes it clear that *E*_0_ = 2 in the baseline region, where *y* is small. One can accommodate variable *E*_0_ by (a) replacing 2 with *E*_0_ in this expression (Mode a), or (2) scaling the entire quantity in square brackets by *E*_0_/2 (Mode b). I have tested both modes, which I will refer to as CMa and CMb. 

Carr and Moore provided the KaleidaGraph routine they used to analyze their data with Equation (10). However, this routine does not properly implement the recursion relation; rather, it just predicts *y_i_* from the *experimental y_i_*_−1_. Accordingly, it contains just the two parameters, *A* and *K_d_*. A true recursion implementation starts with *y*_0_ as a third parameter and generates a full set of *y* values, which are then altered iteratively with adjustment of all three parameters in a nonlinear LS routine. (The FORTRAN function routine for doing this is included in the Supplementary Material.) I have tested the model in this way, with a 4th adjustable parameter for *E*_0_. The method used by CM does converge readily and can provide good initial estimates of the parameters apart from *y*_0_, as needed for the full calculation.

### 2.3. Baseline Functions

The choice of function for base(*n*) can depend on the range of early cycles included in the fit. I have commonly used linear and quadratic functions of *n*, in which the need for parameters beyond the minimal single constant is judged by their statistical significance in the fit. Any parameter having SE greater than its magnitude is statistically undefined in ad hoc fitting [26]. Baselines such as the two from [11] shown in Figure 1B exhibit “saturation” behavior, so are represented as

base(*n*) = *a* − *q* exp(−*rn*) (11)

sometimes with an added linear term. The other baseline in this figure is represented as a constant, after deletion of the first four or five cycles.

### 2.4. Least-Squares Fitting

The NLS fitting was done using routines such as those described before [12,13,14,15]: KaleidaGraph for preliminary work and for the preparation of figures, in-house FORTRAN codes similar to those in [26] for production work on the multireplicate datasets. Weighted fitting was discussed in [15]; although weighting is less important in fitting growth profiles than for *C_q_* calibration data, I have used the same weighting here for consistency. 

In assessing the quality of LS fits, important metrics are the sum of weighted squared residuals, *S* = Σ *wiδi*^2^, and the estimated variance for unit weight, *s_y_*^2^ = *S*/*v*, where the number of statistical degrees of freedom *v* is the number of fitted points minus the number of adjustable parameters. In typical fits of qPCR rx data to the models under investigation here, the model will begin to fail as the number of cycles included in the fit increases, and this failure will manifest as a pronounced rise in the fit variance *s_y_*^2^.

For readers who code, I have a useful tip. Parameters such as *y*_0_ (and to a lesser extent, *y*_max_) can vary considerably—for example, by an order of magnitude with dilution change for 10-fold dilutions. The annoying nonconvergences of NLS fits can be greatly reduced by representing *y*_0_ through its logarithm, e.g., *y*_0_ = 10*^d^*, with *d* now being the adjustable parameter [27].

## 3. Results and Discussion

As Figure 1 shows, the amplification profiles vary considerably over the six datasets, with some having highly symmetrical growth regions (3 × 5 from [18]) and some quite asymmetrical (94 × 4 from [11]). Some develop level plateaus, while others do not. The baselines vary from constant or sloping, to the saturation form of Equation (11). All of these properties can affect the quality of the NLS fits to the models being compared here.

### 3.1. The 3 × 5 Data

I start with and devote much detail to the 3 × 5 data from [18], for which the high symmetry favors the LRE model. As noted earlier in connection with Figure 2, both the LRE and CM models predict realistic transition from *E*_0_ in the baseline region to *E* = 1 in the plateau. However, to prepare this illustration, I had to freeze *E*_0_ in the CM fit at the LRE value; when freely fitted, it was 2.10(11) [≡2.10 ± 0.11] in Mode a and 2.17(7) in Mode b. The *S* values (unweighted fitting) were 1.85 × 10^4^ and 1.36 × 10^4^, respectively, as compared with 1.26 × 10^4^ for the LRE model (with one fewer parameters). With the addition of the asymmetry parameter *p* to the LRE model, the estimated *E*_0_ was 2.066(33) for LREA (*S* = 5237), 1.988(13) for LREB (*S* = 4130), and 1.939(10) for LREC/D (*S* = 7399). Only the last value is within error of the calibration result (1.916(13) from [15]), and it is the statistically poorest of the LRE4 fits. 

**Figure 3:**
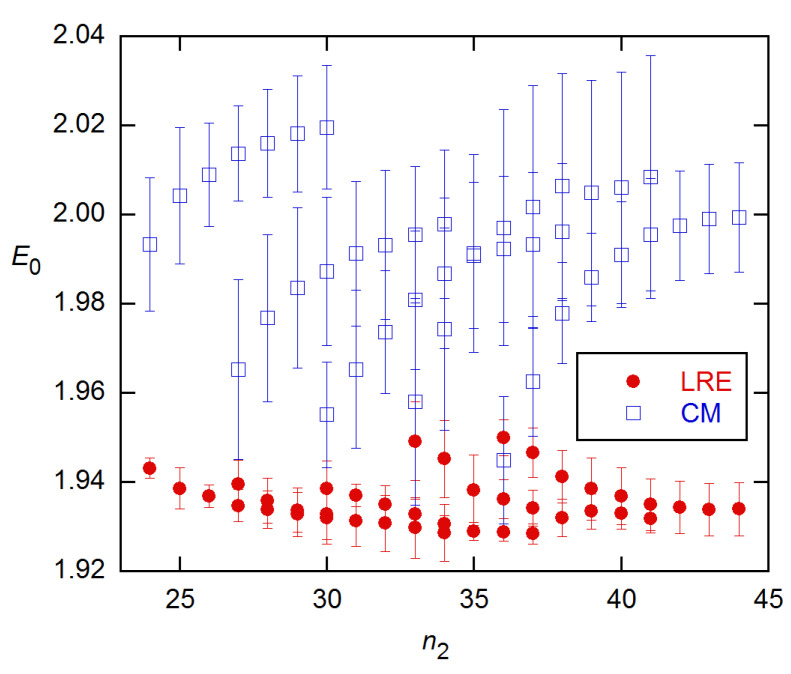
Estimates of initial AE values for the 5 concentrations of the 3 × 5 data from [18], obtained using the LRE and CMa methods and weighted fitting from *n*_1_ = 4 to *n*_2_. Each value is an average of the 3 replicates at that concentration, with error bars being standard deviations (SD). Both methods assumed a constant baseline and used the weighting from [15]. The calibration-based AE is 1.916(13).

Figure 3 compares the CMa and LRE methods on the full 3 × 5 dataset, now giving averages and standard deviations (SD) for the three replicates at each concentration. The CMa *E*_0_s are lower with weighted fitting but are still about 0.1 above the calibration estimate, while the LRE estimates are within about 0.02 of calibration and more precise. 

**Figure 4:**
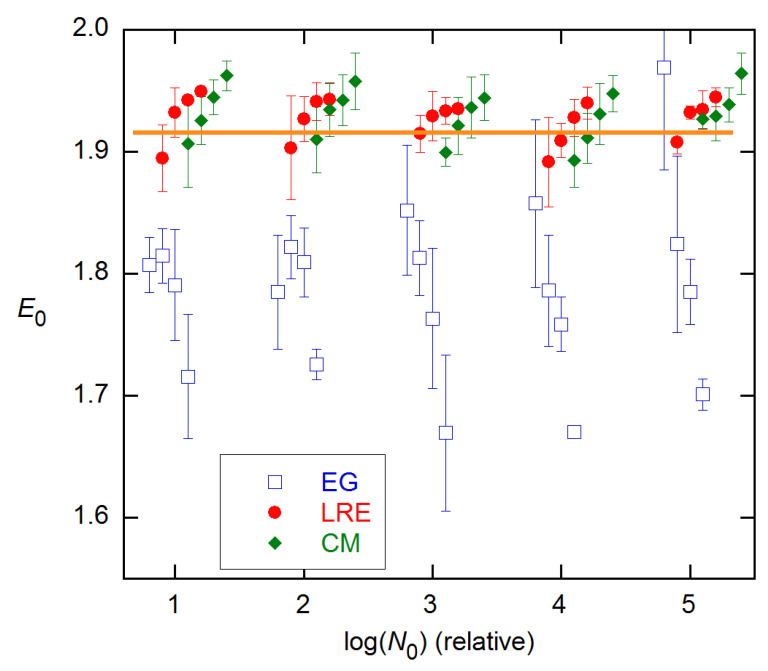
*E*_0_ estimates (averages of 3 replicates) for the 3 × 5 data from [18], obtained for *n*_2_ values near the FDMs for LRE and CMa and the SDMs for EG. Specifically, the LRE estimates start one cycle below the FDMs, the CM one above, and the EG two cycles below the SDMs. With decreasing concentration, the FDMs are 18.5, 22.0, 25.5, 29.3, and 32.4; the SDMs are ~2 cycles lower.

Both models fit the 3 × 5 data well for all n_2_, with the variances in these weighted fits varying with n_2_ by only a factor of ~2, CMa’s being larger than LRE’s by less than a factor of 2. This is in contrast with the behavior for the other datasets, where the variances usually rise sharply around the FDM (see below). Accordingly, I show in Figure 4 the *E*_0_ estimates for *n*_2_ near the FDM, along with EG *E* estimates for *n*_2_ near the SDM. The latter exhibit the behavior predicted by the modeling in [14]: negative bias, minimal near the SDM, with precision increasing with *n*_2_. The CM and LRE estimates agree well with the calibration-based estimate; however, the CM calculations failed to converge for *n*_2_ smaller than ~2 cycles above the FDM, so these CM results are for the lowest converged *n*_2_ values.

In the weighted fits of these data, CMb and CMa gave nearly identical results for both *E*_0_ and *s_y_*^2^ in the range depicted in Figure 4, but the CMb *E*_0_s rose to ~0.01 higher for the largest *n*_2_s covered in Figure 3. The *E*-recursion method of Equation (9) was tried on the data for the highest concentration in this set and gave *E* estimates close to those from CM.

**Figure 5:**
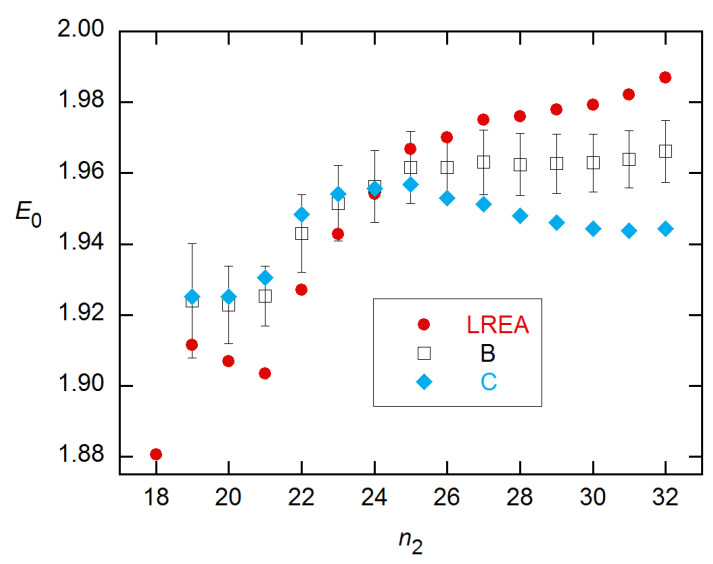
*E*_0_ estimates from the LRE4 models, obtained for the stnd1 data from [18] as a function of *n*_2_. Error bars are SEs from the weighted NLS fits and are comparable for all modes but are shown for only Mode B. Convergence problems limited results at small *n*_2_ to about the FDM (18.5).

However, these were much more disperse, with SDs four times larger than for CM. This greatly reduced precision (and even poorer performance on the 94 × 4 data (below)) led me to drop further consideration of this model. The LRE4 models gave generally lower *E*_0_ estimates than CM for all covered *n*_2_, with the estimates for *n*_2_ < 22 almost all falling within 0.01 of the calibration value (Figure 5). The values for the parameter *p* (Equations (5)–(8)) were mostly close to 1.0 and within their SEs of this value for the lowest several *n*_2_ values, which means the LRE model is statistically better in this region [26].

The mentioned convergence problems occurred for the CM methods for *n*_2_ < FDM + 1 and for LRE with *n*_2_ < FDM − 3. Convergence can be achieved for lower *n*_2_ by freezing a parameter. For LRE, the obvious choice is *y*_max_, and for *n*_2_ < FDM the results are not very sensitive to the value chosen, so values obtained for higher *n*_2_ can be used or even just the approximate plateau value if the plateau is achieved. For the CM method, either *K_d_* or *A* may be frozen, but unfortunately, both begin to change just as convergence becomes problematic. Moreover, these parameters are not related to the plateau in an obvious way; in particular, *A* is about three times the *y*_max_ value. However, I have obtained reasonable *E*_0_ by freezing *A* at either the trend from large *n*_2_ or at the values for nearby *n*_2_, where *A* becomes increasingly uncertain. Since the plateau value can be obtained by simple inspection of the data, the LRE method is easier to use in this way.

### 3.2. Other Datasets

**Figure 6:**
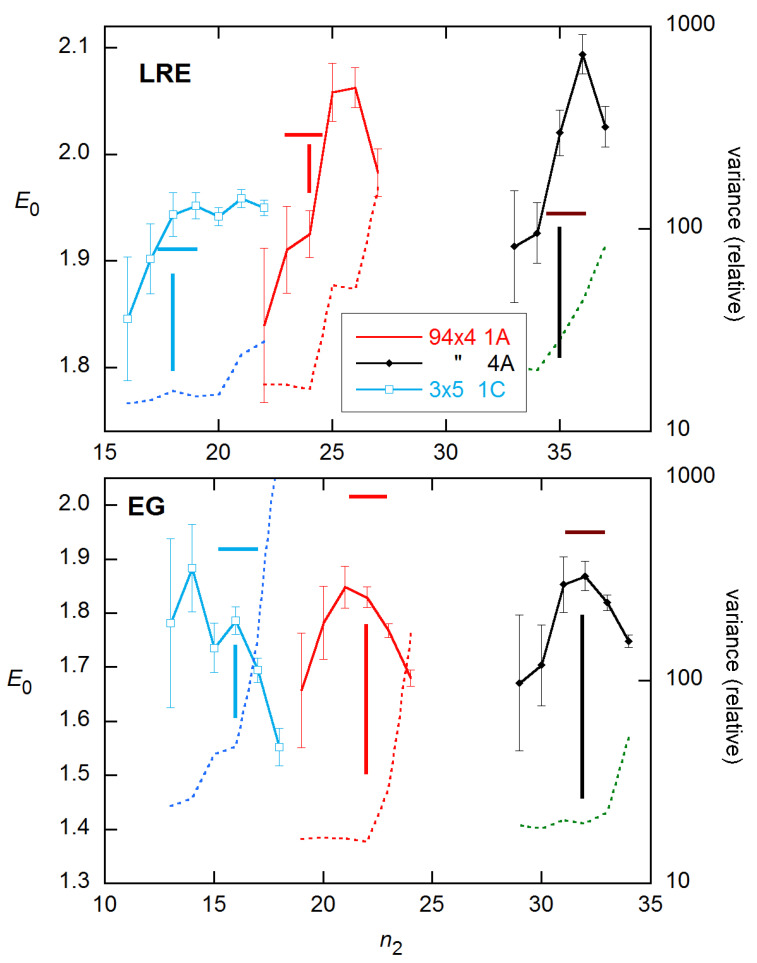
*E*_0_ estimates and fit variances (dashed lines, axes right) for 3 of the reactions shown in Figure 1, obtained using unweighted NLS with the LRE model (**top**) and the EG model, for the indicated *n*_2_ values. Error bars are SEs from the fits. Vertical lines mark the FDMs (**top**) and SDMs (**bottom**); horizontal lines indicate the calibration *E* values.

**Figure 7:**
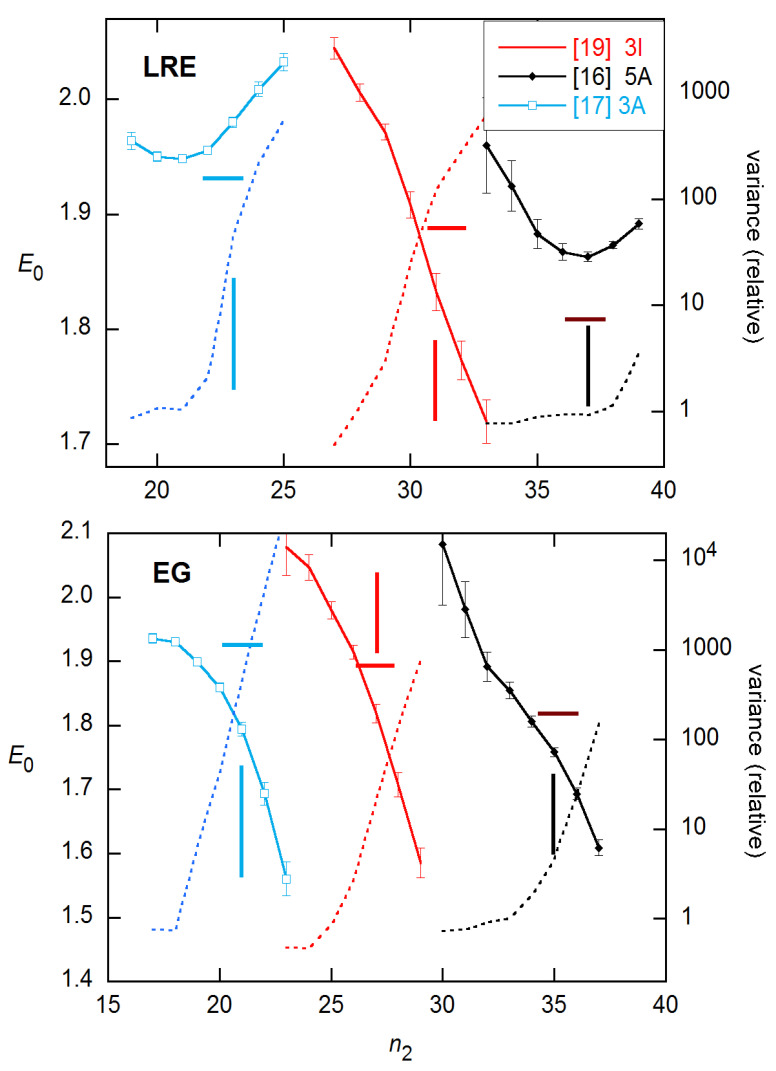
*E*_0_ estimates and fit variances for the other 3 reactions shown in Figure 1, obtained and labeled as in Figure 6.

For the 3 × 5 data examined above, the LRE model performs best and is easiest to employ of the three- and four-parameter methods. Accordingly, in Figure 6 and Figure 7, I compare this method with the EG method that has dominated the literature in various forms, on the representative reactions shown in Figure 1. The LRE results are obtained for *n*_2_ near the FDM cycle, while the EG results center on the SDM cycle. From inspection, the closest agreement with calibration *E* estimates occurs about one cycle below these references in both cases. Comparing statistics for the two methods at these *n*_2_ values, the LRE mean discrepancy is only −0.004, while that for EG is −0.082 (with only one positive difference). The rms differences are 0.053 for LRE and 0.112 for EG. 

**Figure 8:**
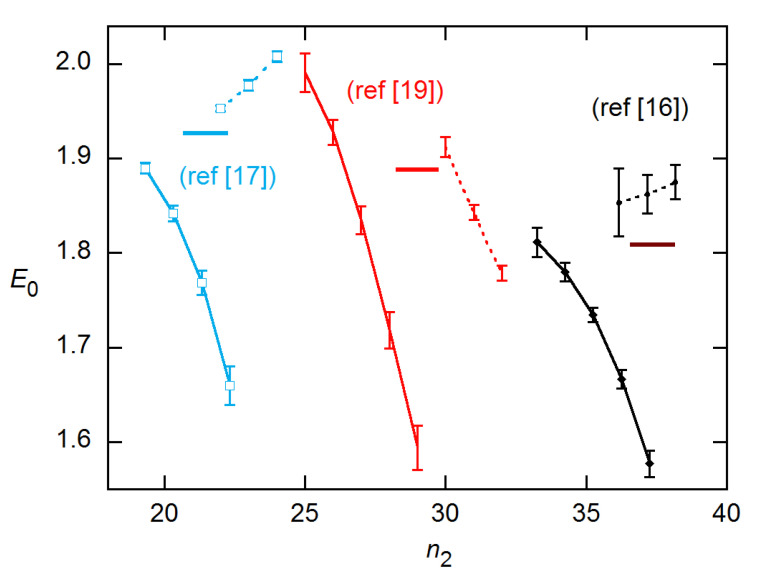
Ensemble results for datasets and concentrations in Figure 7. EG—solid curves; LRE—dashed. Error bars are ensemble SDs.

Although the results shown in Figure 6 and Figure 7 are for single reactions, the ensemble statistics for replicates closely resemble them, as is shown in Figure 8 for the datasets and concentrations of Figure 7. The ensemble SDs of Figure 8 do exceed the SR SEs of Figure 7 in several cases. However, the excess is not as great as found for the *C_q_* estimates in [15].

**Figure 9:**
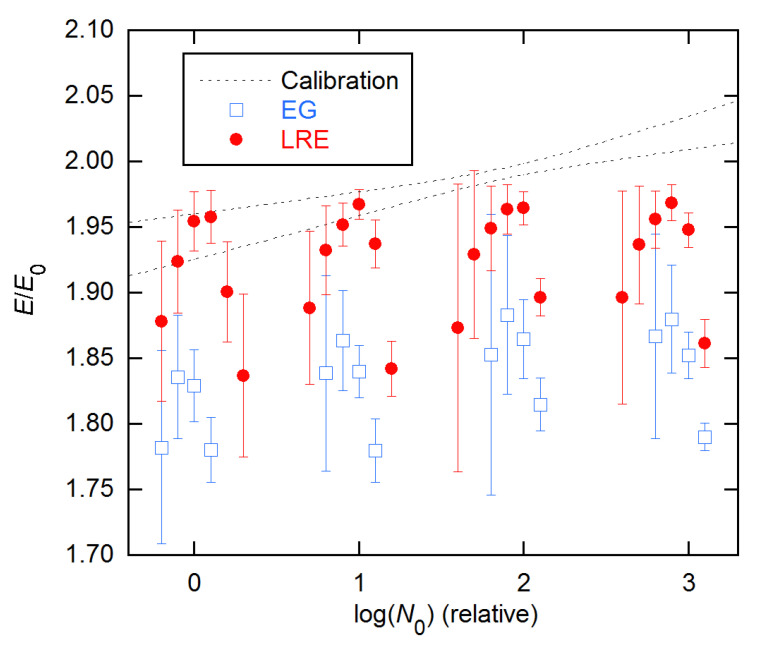
Ensemble AE results for EG and LRE methods on 94 × 4 data from [11], compared with the calibration error band (1 σ) from [15]. EG and LRE estimates are displayed for *n*_2_ values near the SDM and FDM, respectively, with integer values of the abscissa representing those references. In the LRE calculations, *y*_max_ was fixed at the *y* value of the last cycle for each rx.

LRE and EG estimates are compared with the calibration results for all concentrations of the 94 × 4 data in Figure 9. Here, again the EG estimates systematically undershoot the calibration values, while the LRE values agree at the lower concentrations but fall short for higher. These estimates were obtained by fixing the *y*_max_ values; this will be discussed further below. 

**Figure 10:**
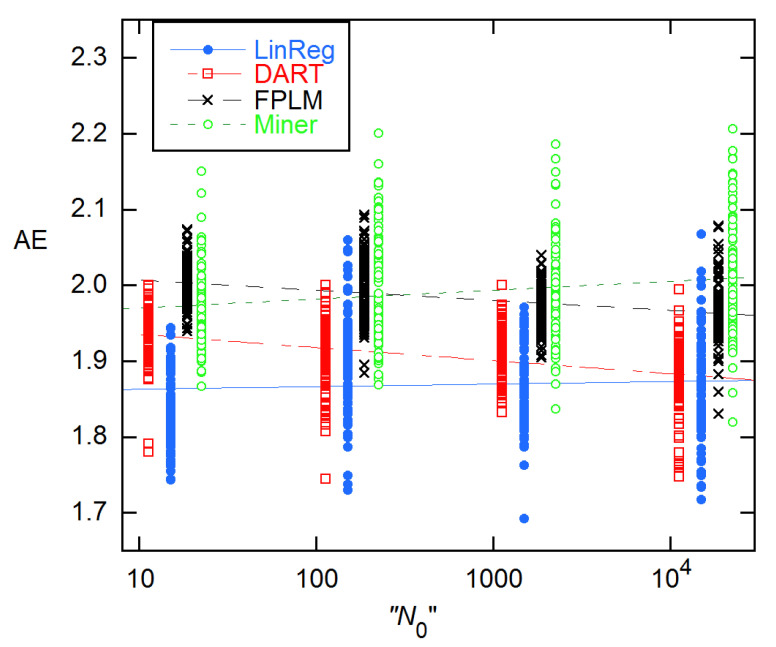
AEs for 94 × 4 data from [11] for four methods that use Equation (1) in various ways. The LinReg values are displayed at the given *N*_0_, while the others have been displaced by ~25% from one another to facilitate display. The lines are LS fits to a straight line in log(*N*_0_); the slopes are significant in all cases, though only marginally so for LinReg. The quotation marks on the *n*-axis label are to indicate that the actual concentrations are the reverse of the labeling given in the Excel data file from [11].

It is instructive to compare the EG results with those given in the supplement to [11], where four of the discussed methods employ the EG model in different ways, two using direct fitting, two using logarithmic transformation, and three subtracting an estimated baseline before fitting. As Figure 10 shows, the results are quite different, giving average AEs ranging from 1.869(62) for LinReg to 1.991(75) for Miner. All sets show a statistically significant slope in log(*N*_0_), and two—LinReg and Miner—support a quadratic dependence.

**Figure 11:**
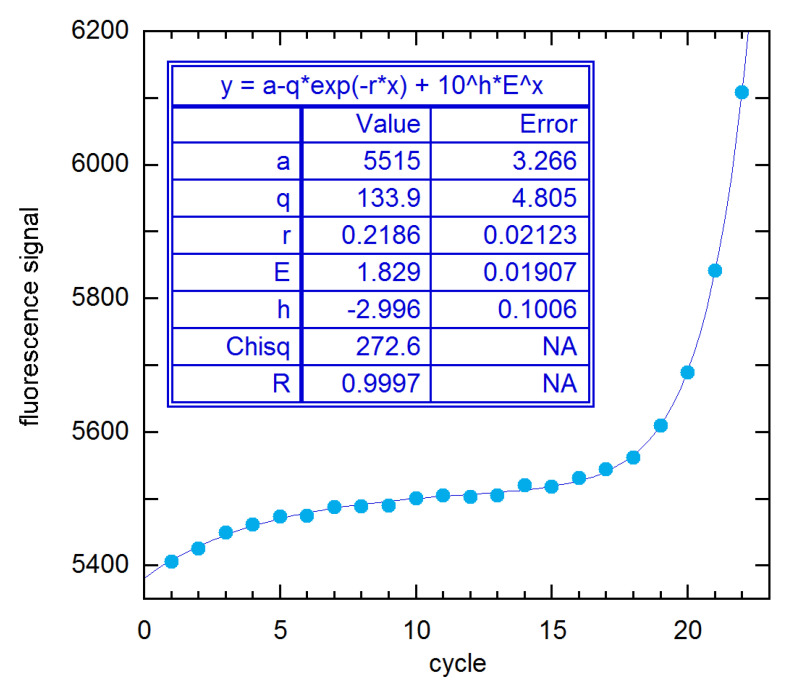
NLS fit of 94 × 4 rx 1A data to the EG model with saturation baseline, from *n*_1_ = 1 to the SDM (*n*_2_ = 22). For this unweighted fit Chisq is *S*, and h is log(*y*_0_).

In an attempt to understand how such ostensibly similar methods can give such disparate results, I have examined results for the 94 × 4 1A rx (MYCN_STDA15–1) of Figure 1 in detail. Figure 11 shows the five-parameter fit of all cycles up to the SDM (22) to Equation (1) plus saturation baseline (Equation (11)). The *E* estimate is smaller than those given for all of the methods shown in Figure 10 and reaches its maximum value (1.848(38)) when *n*_2_ = 21. This is closest to the 1.884 for this rx from LinReg (supplement to [11]), which subtracts a constant baseline and fits a limited range of early growth channels to a straight line after logarithmic transformation. Using the baseline values provided by the authors of this method, I was able to verify their *E* estimates by trial-and-error variation of the fitted cycle range, for example, Cycles 16–22 for the 1A rx. The DART and FPLM methods both subtract a saturation baseline obtained by fitting Cycles 2–10 to Equation (11). That procedure yields a baseline close to that given by the first three parameters in the fit results box in Figure 11. Then FPLM fits the baseline-corrected cycles *n*_1_-SDM to Equation (1) plus a constant, where *n*_1_ is determined from statistical tests. The result in [11] is 2.012. For *n*_1_ = 11–18 and *n*_2_ = 22 (the SDM), this procedure yielded a maximum *E* of 1.800(18) in my calculations. By reducing *n*_2_, I found a maximum *E* of 1.95(10) for Cycles 16–20. Similarly, the Miner method fits to the same 3-parameter expression, but without first subtracting a saturation baseline. The largest *E* I could get this way was 1.93(10) fitting Cycles 16–20, as compared with the Miner estimate of 1.992. While the discrepancy is within statistical error for these five-cycle fits, both methods claim to fit cycles to the SDM, and there the differences are clearly an irreconcilable 0.2 in magnitude. As a further illustration of such baseline and cycle-range problems, I note that adding a baseline slope to the Miner procedure gives *E* = 1.986(63) fitting Cycles 9–21, as compared with a maximum of 1.900(34) for *n*_2_ = 21 and any *n*_1_, with a constant baseline. 

**Figure 12:**
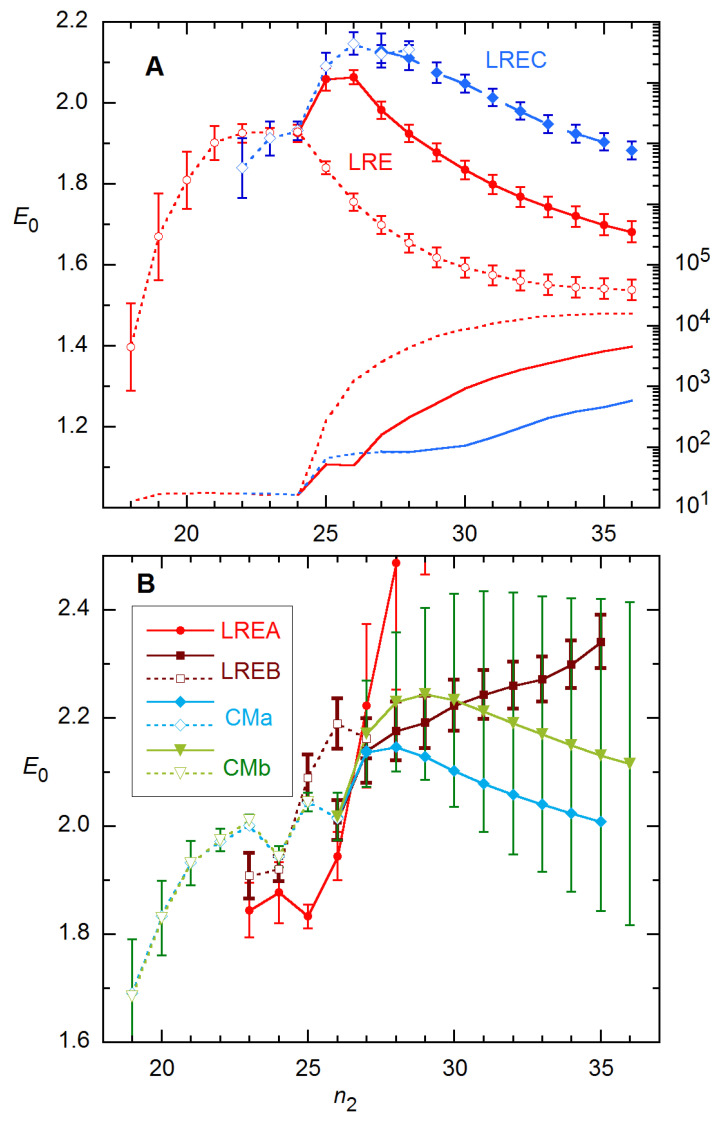
*E*_0_ and fit variance for 94 × 4 rx 1A analyzed as a function of *n*_2_ for 4 LRE (**A**,**B**) and 2 CM modes (**B**). Solid curves and points indicate that all parameters were fitted, while dashed curves and open points represent results obtained with parameters frozen—*y*_max_ for the LRE models, *K_d_* for CM and then *A* also below *n*_2_ = FDM (24). The fitted cycles start with *n*_1_ = 1, and the saturated baseline function is used. Error bars are estimated SEs from the fits; for CMa (not shown), they are comparable to those for CMb.

Results for this rx obtained with the four-parameter models are shown in Figure 12. For estimating *E*_0_, the best performers are the simple LRE model (i.e., *p* = 1) and LREC, illustrated in Frame A. The fit variance rises sharply beyond *n*_2_ = 24 (the FDM) for all CM and LRE modes except LREA (*n*_2_ = 25), indicating that the fit models are inadequate over cycle ranges extending beyond these *n*_2_s. Further, for all methods except LRE and LREA, I had to freeze at least one parameter in order to achieve convergence for *n*_2_ ≤ 24. For the CM methods, this requires fitting the data for larger *n*_2_ to arrive at appropriate values for the frozen parameter(s). Figure 12A includes results obtained for LRE at all *n*_2_ with the plateau level frozen at the value of *y* for the last cycle (making *y*_max_ the difference between this *y_i_* value and the baseline *a*). 

It is noteworthy that the *E*_0_ estimates at *n*_2_ = 24 are the closest to the calibration value for most variations on these methods: 1.925(22) for LRE, 1.88(6) for LREA [1.94(4) at *n*_2_ = 26], 1.921(23) for LREB, 1.926(22) for LREC, 1.945(19) for both CMa and CMb [1.97(2) at *n*_2_ = 22]. These values all undershoot the “true” *E*_0_ = 1.99 but are mostly within ~0.05. For reference, the FDM for this profile is 24.4. So again, here, the best estimates of *E*_0_ and the rise in variance occur near the FDM.

A few additional details of these calculations warrant mention. (1) For most of the *E*_0_ results stated just above, at least one parameter had to be held fixed in the fit—*K_d_* for the CM estimates (and *A* at *n*_2_ = 22), *y*_max_ for LRE4. (2) While the *E*_0_ parameter correctly describes *E*(*n*) in the baseline region for all models, *E*(*n*) does not go properly to 1 in the plateau region for LREC and LRED. (3) Correspondingly, *y*_max_ in the expression for LREC (Equation (7)) and *n*_FDM_ for LRED depart from their “true” values with increasing *n*_2_. (4) The SE error bars for the CMb method in Figure 12B are greatly pessimistic, as the ensemble SDs are close to those for the LRE4 methods for both CM modes. (5) The *E*(*n*) recursion method of Equation (9) performs poorly on these data, giving *E*_0_ > 2.5 with SD > 0.15 for data at the highest concentration. With regard to point (1), all parameters can be fitted for LRE; and for LREB and LREC, *E*_0_ is insensitive to the fixed plateau, changing by <0.01 for ±1000 change from the actual plateau of ~10500.

**Figure 13:**
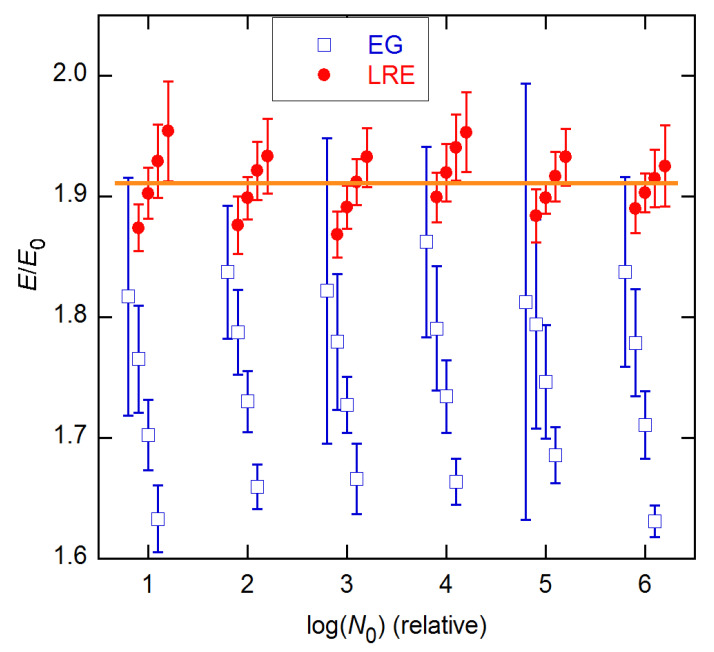
Ensemble AE results for EG and LRE methods on 20 × 6 data from [3]. The horizontal line indicates the calibration estimate from [15]. The EG and LRE estimates are displayed for *n*_2_ values near the SDM and FDM, respectively, with integer values of the abscissa representing those references. In the LRE calculations, *y*_max_ was fixed at the value determined from fits to the LL4 model of Equation (4).

LRE and EG results are compared for the data from Rutledge and Cote [3] in Figure 13. The amplification profiles are fairly symmetrical for these data, and again the LRE results agree well with calibration, while EG fall below. Similar results are shown for the datasets from [16], [17], and [19] in the online supplement. For two of these, the *C_q_*-based calibration estimates are themselves uncertain, as the dependence of *C_q_* on log(*N*_0_) is not linear, and the AE values vary significantly with the fit order [15].

From these examples, we see that both the CM and LRE methods *can* give reasonable estimates of *E*_0_ for both symmetrical and unsymmetrical growth profiles. For the latter, however, practical problems—especially convergence difficulties and for CM, problems choosing the values for frozen parameters—make implementation difficult for large-scale applications processing many profiles without operator intervention. The simplest approach is just the LRE method (i.e., *p* = 1) with *y*_max_ either fitted or determined by *y_i_* for the last cycle: As the fitted estimates of *y*_max_ become progressively more uncertain with decreasing *n*_2_, the so-fixed *y*_max_ values are generally within the statistical uncertainty given by the parametric SE, and the *E*_0_ estimates are not very sensitive to this choice anyway. 

As was noted in the discussion of Figure 6 and Figure 7, overall, the best EG *E* estimates come from *n*_2_ = SDM – 1, and the best LRE *E*_0_s come from *n*_2_ = FDM − 1, where the computations generally converge easily. With the latter, freezing *y*_max_ at either the last *y_i_* or the estimate from a fit of the data in the transition region to the LL4 model of Equation (4) sometimes lowers the estimated *E*_0_, and it sometimes raises it. For the six datasets examined here, freezing *y*_max_ improved agreement with calibration for three datasets, but worsened it for the other three. 

## 4. Conclusions

The standard approaches for estimating qPCR amplification efficiency from single-reaction data have relied on the two-parameter exponential growth model of Equation (1), with various ways of treating (and usually subtracting) the baseline and selecting the cycle range for the AE estimation. These methods have previously been shown to lead to bias and loss of precision. In this work, I have examined several models that allow for reasonable decline of *E*(*n*) in the growth region: (1) the three-parameter LRE model [21,24], (2) four four-parameter extensions of LRE that preserve the physical significance of *E*_0_ as the AE through the baseline region, (3) two four-parameter modifications of the recursion method of Carr and Moore [22] that also permit estimation of *E*_0_ ≠ 2 in the baseline region, and (4) a four-parameter recursion method that fits directly to a sigmoidal *E*(*n*) function. All but the last of these can give good estimates of *E*_0_, and for the six multireplicate datasets considered here, give results closest to the calibration-based AE estimates when the data are fitted to about one cycle below the FDM. For such small *n*_2_, the four-parameter methods can give convergence problems, necessitating that at least one parameter be held fixed. 

For the six multireplicate datasets tested here, the LRE method rarely presented such problems. Since, overall, its *E*_0_ estimates were as good as those from the four-parameter methods, my presently recommended procedure is to fit the three-parameter LRE model to data extending to one cycle below the FDM. Importantly, a suitable baseline function must be included in the fit model for optimal performance. The resulting four–six parameter models are easily handled by readily available nonlinear least-squares algorithms.

Given that the SR methods are destined to remain uncertain in their estimation of *E*_0_ for new systems, it may be necessary to benchmark them against standard calibration methods for similar systems and experimental procedures. Of course, such corrections can also be used for the EG method, which, however, is typically less precise near *n*_2_ = *n*_SDM_ and varies more with a change in *n*_2_. It is also useful to consider how much error can be expected and/or tolerated in SR estimates for unknowns. This error will depend upon how far removed the unknown is from some known reference, as given by Δ*C_q_*. If the *E*_0_ estimate is off by 5% (0.1 for *E*_0_ ≈ 2), the error will be about *E*_0_^0.05 Δ*Cq*^, which, for example, is +65%/−40% for Δ*C_q_* = ±10. From the present work, 5% error in *E*_0_ is a conservative estimate of the LRE reliability. It was only clearly exceeded for the data from Guescini et al. [17] (see supplement), where the estimates exceeded 2.0 and so would be reduced to that value in recognition of the physical limit on *E*_0_.

## Data Availability

Any datasets that are no longer available from the cited data sources may be obtained at www.dr-spiess.de, accessed on 13 July 2021.

## Supplementary Materials

The following are available online at https://www.mdpi.com/article/10.3390/life11070693/s1, includes three Figures S1–S3 comparing EG and LRE results for the datasets from refs [16,17,19], and two Figures S4 and S5 giving FORTRAN function routines for the two recursion models examined in this work.
