# Supplementary material for: Estimating Real-Time qPCR Amplification Efficiency from Single-Reaction Data
Source: Life (Basel). 2021 Jul 14;11(7):693. doi: 10.3390/life11070693 (PMC8303528; doi:10.3390/life11070693)
Supplement: Supplementary file 1 [file life-11-00693-s001.zip › life-1272248-supplementary.pdf]

Online Supplement to

Estimating real-time qPCR amplification efficiency from single-reaction data

by

Joel Tellinghuisen, Department of Chemistry, Vanderbilt University
Nashville, Tennessee, USA 37235

I provide here (1) LRE/EG comparison results for the datasets from Guescini, *et al.* [17], Lievens, *et al.* [19], and Karlen, *et al.* [16]; and (2) listings for short FORTRAN routines for the $E(n)$ and Carr-Moore recursion methods. Reference numbers are for citations in the paper.

The Guescini results are shown in Figure S1. As in other cases, the EG results are systematically low for n_2 = SDM but do agree better for smaller n_2. The LRE fits with $y_{\max}$ fitted rather than frozen give an order-of-magnitude smaller S and s_y^2, leading to smaller individual fit SEs and also to tighter grouping of the 12 replicate estimates at each concentration. Freezing $y_{\max}$ at the last y_i value makes little difference for the red points, as the plateaus are flat, giving values close to the LL4-fitted $y_{\max}$ values. The LREC method was also run on these data, with fixed $y_{\max}$, giving E_0 values close to the green points in Figure S1.

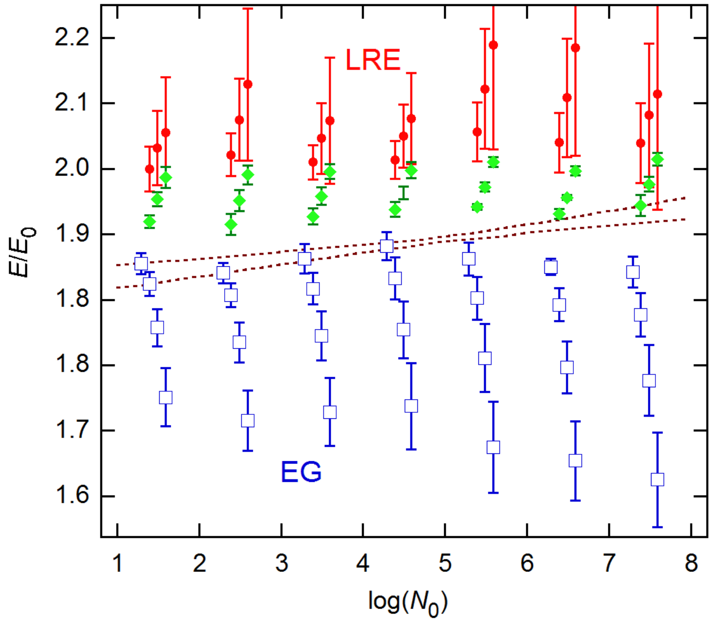

Figure S1. E estimates for data from [17] from the EG method (blue) and E_0 from LRE with $y_{\max}$ fitted (green) and fixed (red) at the value from a fit of 21 points centered on the FDM to the LL4 model of Eq. (4). The dashed lines represent the 1-σ error band on E from the quadratic calibration fit in [15]. Displayed EG values are obtained fitting to n_2 = SDM−2 to SDM+1, LRE from FDM−1 to FDM+1. Error bars are ensemble SDs. (FDMs are approximately 16, 19, 23, 26, 30, 34, and 37; SDMs fall about 2 cycles lower.)

Results for the Lievens data [19] are shown in Figure S2. The dependence of C_q on $\log(N_0)$ is not linear, so calibration-based estimates are shown for representative fits of comparable statistical quality [15]. The point is to indicate that the standard approach yields only rough estimates in this case — ~1.65 for the AE at low concentrations, rising to ~1.9 at high. None of the SR methods gives estimates that track this increasing trend, with the "blue" LREs being arguably the poorest and the "red" LREs the best. As in Figure S1, freezing y_{max} led to an order-of-magnitude increase in the fit variances. (To limit display congestion, estimates for multiple adjacent n_2 values are shown at only one concentration for each of the SR methods.)

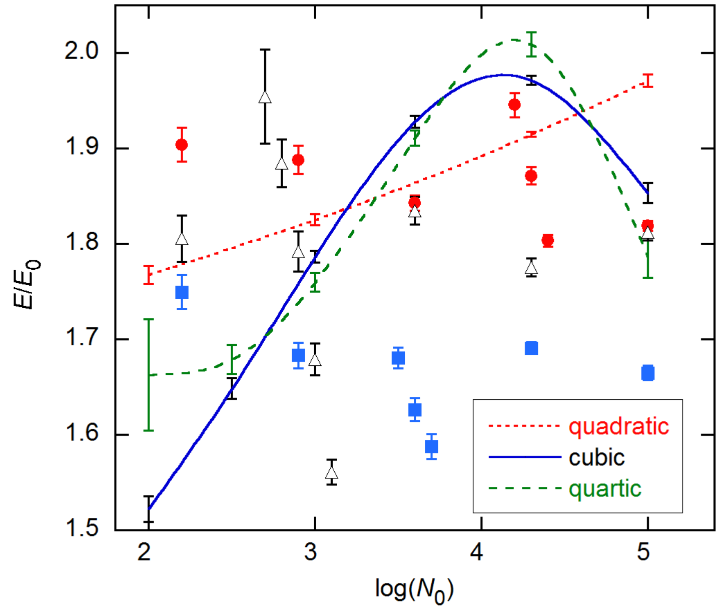

Figure S2. *E* estimates for data from [19] from the EG method (triangles) and E_0 from LRE with y_{max} fitted (red) and fixed (blue) as in Figure S1. Three calibration-based *E* estimates from [15] are included. The LRE E_0s are displayed at the FDMs — n_2 = 26, 28, 31, 33, 36 — and the EG *E*s at the SDMs (about 3 cycles smaller). Values for additional n_2 differing by ±1 are shown for LRE at the 3rd and 4th concentrations, with 4 adjacent n_2 values for EG at the 2nd concentration.

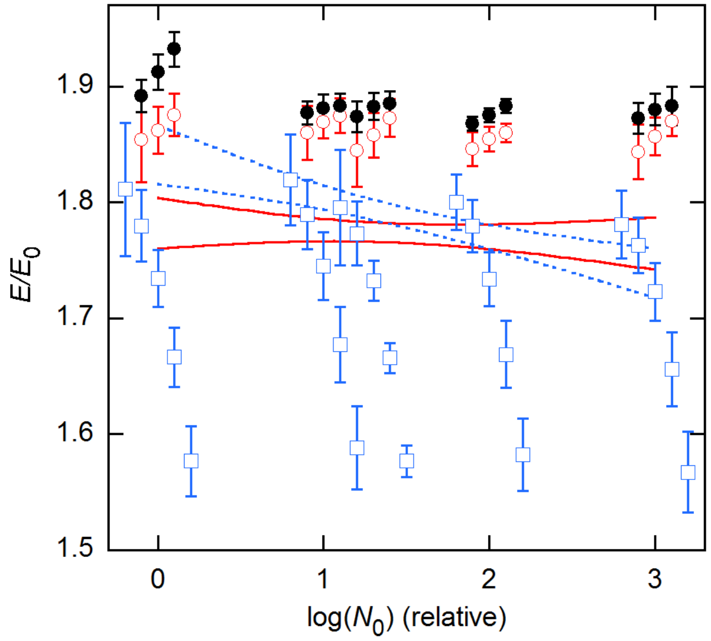

Figure S3. *E* estimates for data from [16] from the EG method (blue) and E_0 from LRE with y_{max} fitted (red) and fixed (black) as in Figure S1. The quadratic calibration *E* error bands from [15] are for two C_q markers, SDM (red) and relative threshold C_r. The diplayed LRE points are for n_2 = FDM ± 1, and the EG points are centered at n_2 = SDM. (FDM *n*s are approximately 25, 29, 32, 34, 37, and the SDMs ~2 cycles smaller.)

Figure S3 displays results obtained for the data from Karlen, *et al.* [16]. Here again the standard C_q calibration approach fails to produce definitive results, with the two illustrated E error bands coming from quadratic fits of C_q vs. $\log(N_0)$ for two different C_q markers. Allowing for this uncertainty, this dataset is the single case among the six investigated here where the EG estimates are better than the LRE, with the Es at SDM−1 in essential agreement for all concentrations.

I show below in Figures S4 and S5 the function routines used in the CM and E-recursion programs. Although these are in the "prehistoric" FORTRAN language, the operations are easy to follow, so easily translated to other programming languages. Unfortunately, I have not been able to determine a way to make them work in the KaleidaGraph General routine. It appears possible to implement them there through a manual iterative process; but given the very large number of iterations I have often found necessary to achieve convergence, this would be extremely tedious.

```
      DOUBLE PRECISION FUNCTION FUNC(N,C,NV,X,KK)
      IMPLICIT REAL*8(A-H,O-Z)
      DIMENSION  C(N),X(900,6)
          NOVAR = NV
          T = X(KK,1)
          NCYC = T + .1
          DK = 10.**C(2)
          A = 10.**C(3)
          D2 = 10.**C(1)
      DO 10 I = 1,NCYC
          D1 = D2
          TERM = C(7) - D1*(1./A + 1./(DK+D1))
C          TERM = 2. - D1*(1./A + 1./(DK+D1))
        IF (TERM.LT.0.0) TERM = -TERM
          D2 = D1*TERM
C          D2 = D1*TERM*C(7)/2.
   10 CONTINUE
          BASE = C(4) - C(5)*EXP(-C(6)*T)
C          BASE = C(4) + C(5)*T + C(6)*T*T
          CALC = D2 + BASE
      FUNC = CALC - X(KK,2)
      RETURN
      END
```

Figure S4. FORTRAN FUNCTION routine for the CM program. The data are passed through the `X(I,J)` variable, with `J=1` for cycle number and `J=2` for fluorescence signal. This routine is called from the main program pointwise, with `I=KK`. The first three adjustable parameters are the $\log_{10}$ values of the quantities appearing in Eq. (10) in the paper, with `C(1)` being $\log10(y_0)$. E_0 is `C(7)`. Loop 10 does the recursion, from cycle 1 to `X(KK,1)`. It is set for Mode a, but can be converted to Mode b by moving the "commenting" `C`s to the lines just preceding them. Similarly, the saturation `BASE` can be converted to a quadratic baseline.

```
      DOUBLE PRECISION FUNCTION FUNC(N,C,NV,X,KK)
      IMPLICIT REAL*8(A-H,O-Z)
      DIMENSION  C(N),X(900,6)
          NOVAR = NV
          T = X(KK,1)
          ICT = T + .1
          D2 = 10.**C(1)
          BB = C(2)
          CC = C(3)
          DD = C(7)
      DO 10 I = 1,ICT
          XX = I
          EFF = 1.0 + DD/(1. + EXP(BB*(XX-CC)))
          D1 = D2
          D2 = D1*EFF
   10 CONTINUE
          BASE = C(4) - C(5)*EXP(-C(6)*T)
C          BASE = C(4) + C(5)*T + C(6)*T*T
          CALC = D2 + BASE
      FUNC = CALC - X(KK,2)
      RETURN
      END
```

Figure S5. FORTRAN FUNCTION routine for $E(n)$ recursion. $E(n)$ is defined by the logistic function in Loop 10. Accordingly, E_0 = `DD+1`. The routine is otherwise the same as given in Figure S4.
